# Chronic Cavitary Pulmonary Histoplasmosis in an Immunocompetent Patient

**DOI:** 10.7759/cureus.37095

**Published:** 2023-04-04

**Authors:** Maram Albandak, Jehad Azar, Mohammed Ayyad, Qais Salah, Anas Toqan, Narmeen Giacaman, Nizar Marzouqa, Mohammed Al-Tawil, Bisan Wishah, Anas Barabrah

**Affiliations:** 1 Internal Medicine, Al-Quds University, Jerusalem, PSE; 2 Respiratory Institute, Cleveland Clinic, Cleveland, USA; 3 Epidemiology and Public Health, Western Sydney University, Sydney, AUS

**Keywords:** chronic infection, granuloma, fungal infection, histoplasma capsulatum, pulmonary histoplasmosis

## Abstract

*Histoplasma capsulatum* is a fungal organism that causes systemic histoplasmosis. It is commonly asymptomatic in healthy immunocompetent individuals. The clinical symptoms of chronic cavitary histoplasmosis are typically seen in the immunodeficient population, particularly in smokers with pre-existing structural lung disease. We report a case of chronic cavitary histoplasmosis in an immunocompetent patient from an endemic area without pre-existing structural lung pathology. She presented complaining of right hypochondrial pain and had no history of respiratory symptoms nor history suggestive of immunosuppression, tuberculosis, or recent travel. CT scan revealed a cavitary lung lesion and a hilar mediastinal mass. Biopsies obtained by bronchoscopy revealed signs of necrosis, granulomas, and the presence of fungal organisms consistent with histoplasmosis. *Histoplasma* antibodies by complement fixation for yeast antibodies test were positive establishing the diagnosis of chronic cavitary pulmonary histoplasmosis (CCPH). She was then started on itraconazole with good tolerance. On follow-up three months later, a chest CT done along with measurement of inflammatory markers and liver enzymes demonstrated complete clinical recovery. This case emphasizes the importance of expanding our current understanding of the clinical presentation and manifestations of histoplasmosis beyond the conventional assumption that severe disease only affects immunocompromised individuals.

## Introduction

*Histoplasma capsulatum*, a dimorphic fungus, is the causative agent of histoplasmosis [1]. This fungus is most commonly found in Central and North America, especially in Mississippi and Ohio River valleys [1]. However, it can be found in other various regions around the world [2]. The burden of the disease depends mainly on the amount of microconidia inhaled, and whether the host is immunocompetent or immunocompromised. The scale of symptom occurrence is considerably variable; most healthy, immunocompetent individuals will develop mild respiratory symptoms or even will be asymptomatic. The invasive and chronic forms of histoplasmosis occur usually in immunodeficient patients, and the complications of these forms can be severe and sometimes even fatal [3]. Patients with underlying pulmonary diseases are at increased risk for developing chronic histoplasmosis, which is usually associated with lung cavitations [4]. These cavities are likely to enlarge and involve many areas within the lungs. Affected individuals present with a wide range of respiratory symptoms, including productive cough, shortness of breath, hemoptysis, chest pain, fevers, and weight loss. An uncommon, yet unfortunate complication of histoplasmosis infection is fibrosing mediastinitis: also known as sclerosing mediastinitis, which is identified by extensive fibrotic reaction in the mediastinum [5].

We present a case of chronic cavitary histoplasmosis in an immunocompetent nonsmoker patient in the absence of underlying structural lung disease, who presented with unilateral hypochondriac abdominal pain.

## Case presentation

A female patient in her thirties presented to the emergency department (ED) complaining of right hypochondrial pain for which abdominal and chest CT scans were done and revealed an incidental finding of a cavitary lesion in the lower lobe of the right lung. The patient denied any history of respiratory symptoms or constitutional symptoms (weight loss, loss of appetite, night sweats, fever, or itching). She didn’t have any history suggestive of aspiration, immunosuppression, tuberculous, and other atypical infections, and symptoms suggesting rheumatological, connective tissue, or autoimmune disease. 

The patient also had no dyspnea, cough, expectoration, fever, chills, hemoptysis, or any history of recurrent chest infections. Past medical history was significant for childhood asthma, a mild remote history of coronavirus disease 2019 (COVID-19) infection only involving the upper airways, as well as an episode of viral sinusitis, migraine, hyperlipidemia, obesity, and depression. Past surgical history was non-revealing.

Social history revealed that the patient was not a smoker, had no pets, didn’t drink alcohol or use illicit drugs, and had no recent travel history. Occupational and environmental history was unrevealing. The patient had no family history of any connective tissue disease, malignancy, or other pulmonary diseases. The physical examination was otherwise non-contributory. Vital signs in the ED were within normal limits.

**Figure 1:**
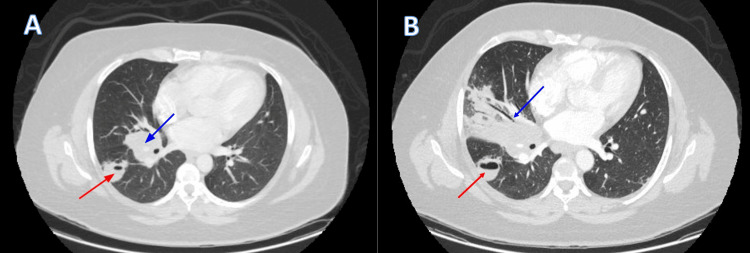
Initial computerized tomography (CT) scans Presence of right hilar lobulated mass measuring 4.5*4.5 cm (blue arrow) and RLL cavitary lesion measuring 2.8*3 cm with air-fluid level (red arrow) in soft tissue window (A) and lung window (B).

**Figure 2:**
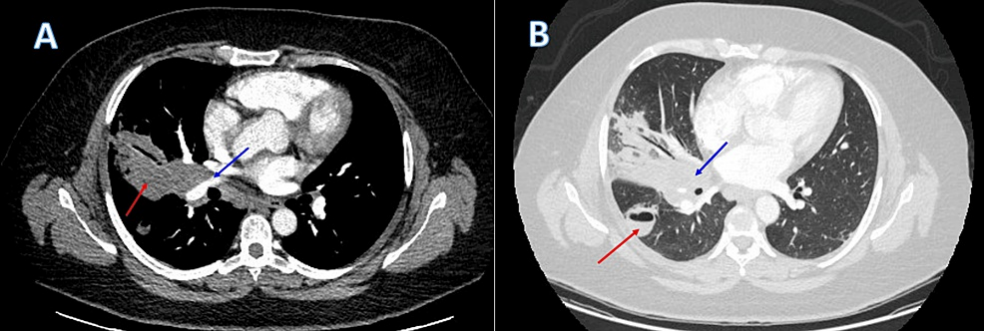
CT scans following bronchoscopy (A) Soft tissue window showing RML consolidation with air bronchogram (red arrow). The proximal anterior RLL pulmonary artery is encased within the right hilar mass and mildly compressed; (B) Lung window showing RLL cavitary lesion with air fluid level (red arrow) and RML consolidation with air bronchogram (blue arrow).

Upon admission, a CT scan of the chest was ordered and revealed a right hilar mass along with right lower lobe abscess, indicating an indolent infection (Figure 1A, 1B). As part of further evaluation, a transthoracic echocardiogram (TTE) was negative for vegetations or valvular lesions ruling out infective endocarditis. In addition, the patient underwent bronchoscopy with bronchoalveolar lavage (BAL), along with endo-bronchial ultrasound with right lung and hilar mass transbronchial needle aspiration biopsies. A few days following discharge, the patient was re-admitted for pneumonia secondary to the bronchoscopy procedure, for which she received a course of oral antibiotics. CT scan done upon readmission showed an interval increase in the size of the right hilar mass, along with post-obstructive consolidation, and volume loss in the right middle lobe (Figure 2A, 2B). Moreover, the cavitary lesion in the right lower lobe (RLL) had increased in size compared to the prior scan, with evidence of constriction in the bronchi of the right middle lobe (RML), and anterior segment of the RLL. Fibrous tissue was also seen encasing the pulmonary arteries, and RML pulmonary vein. Put together, all these radiological findings were suggestive of early signs of fibrosing mediastinitis.

Biopsies obtained from the bronchoscopy procedure were sent for pathology and showed signs of necrosis, fibrosis, and caseating and non-caseating granulomas. The culture was obtained and four weeks later revealed white-yellowish colonies of fungal organisms with a central raised area surrounded by a flat spreading outer zone, consistent with histoplasmosis. A full workup was done including a complete blood count (CBC), C-reactive protein (CRP), rheumatoid factor (RF), antinuclear antibody (ANA), antinuclear cytoplasmic antibodies (ANCA), along with blood, urine, and sputum cultures, which excluded rheumatological and infectious causes. Laboratory and radiological workups for malignancy were also negative. Workup for immunodeficiency was done, including immunoglobulin levels, human immunodeficiency virus (HIV) testing, complement levels, delayed-type hypersensitivity skin testing using *Candida* antigen, and flow cytometry, all of which were within normal limits. In addition, the infectious BAL panel returned negative for viral, fungal, and bacterial causes. Urine and serum *Histoplasma* antigen tests were negative. *Histoplasma* antibodies by complement fixation for yeast antibodies test were positive at 1:64, while mycelial form antibodies were negative. *Histoplasma* antibodies by fungal immunodiffusion were negative too. The biopsy findings combined with the positive *Histoplasma* serology confirmed the diagnosis of histoplasmosis.

The clinical picture as well as the laboratory, biopsy, and imaging findings of the patient led to a diagnosis of chronic cavitary pulmonary histoplasmosis (CCPH). The patient was started on oral itraconazole with good tolerance. Two-week trough level showed a marginal therapeutic level. The dose of itraconazole was increased.

A follow-up CT scan was done after three months and showed gradual resolution of the right hilar lesions and RLL abscess. Repeat measurements of inflammatory markers and liver enzymes were normal. Consultation with the infectious disease department was also undertaken.

On follow-up, she reported complete clinical recovery with no signs or symptoms of pleuritic chest pain, abdominal pain, cough, or expectoration. The patient continued taking oral itraconazole therapy, trough level was always therapeutic. She was advised to continue itraconazole for 12 months with strict follow-up for signs of recurrence upon stopping the medication.

## Discussion

*Histoplasma capsulatum* is a non-encapsulated dimorphic fungus that causes histoplasmosis, a systemic fungal infection. Infection with *Histoplasma* is most prevalent in North and Central America, but the organism also occurs in other diverse areas of the world. In the United States, it is most prevalent around the Mississippi and Ohio River Valleys. Soil containing a great amount of bird or bat droppings, especially next to chicken coops and in caves, remains the main source of *Histoplasma* [1].

Histoplasmosis has been reported in immunocompromised individuals, including those with HIV, diabetes, alcoholics, transplant recipients, and those on immunosuppressive medications and biologics [6]. Only less than 1% of those exposed to the fungus develop clinical disease, depending on the immune status of the patient and the amount of exposure [7].

Infection with *Histoplasma* occurs mainly by inhalation of contaminated soil containing microconidia, the infectious form of *Histoplasma*, in endemic areas during day-to-day pursuits. Once in the lower airways, macrophages ingest the microconidia, which transform into yeast, and multiply inside, then spread by means of the reticuloendothelial system. Antigen-presenting dendritic cells recognize the organism and present it to T-lymphocytes, which stimulates their proliferation and release of cytokines such as tumor necrosis factor-alpha (TNF-a) and interferon-gamma, as well as interleukin 12 from macrophages. The end result is granuloma formation, which helps contain the organism and prevent its dissemination [8]. Thus, any defect in cell-mediated immunity contributes to *Histoplasma* infection. As mentioned, after thorough evaluation, our patient had no evidence of immune deficiency and thus introduced an unusual case of chronic cavitary histoplasmosis in an immunocompetent patient. It is possible that immunocompetent individuals may develop progressive disseminated histoplasmosis due to endogenous reactivation of the infection several years later, similar to what is observed in TB [9].

Data extrapolated from a multistate epidemiological study of histoplasmosis showed that 56% of patients presenting with mildly symptomatic disease were immunocompetent, while only 30% of immunocompromised patients had comparable severity [10]. Cough was the most common presenting symptom in both groups. Furthermore, being in an immunocompromised state was associated with a 78% increase in the likelihood of developing severe disease requiring hospitalization compared to those with immunocompetent status [10]. Interestingly, although being immunocompetent is protective against developing severe histoplasmosis, factors such as age, high dosage of exposure to the organism, and non-White race possibly relating to lower socioeconomic status, poor access to medical care, and genetics, all increase the risk for developing advanced disease requiring hospitalization [10].

Exposure to *Histoplasma* is especially common in individuals residing in endemic areas; however, clinically symptomatic infection is not. A large number of those exposed do not develop symptoms or only develop mild disease not recognized as histoplasmosis [1]. Histoplasmosis can be clinically divided into acute pulmonary infection, chronic cavitary pulmonary infection, extrapulmonary progressive disseminated infection, and mediastinal lymphadenitis (Table 1) [11]. Interestingly, chronic cavitary histoplasmosis has a predilection for older patients and is more likely to occur in smokers with pre-existing structural lung diseases like emphysematous lungs [1,11]. It is strikingly atypical for a patient without pre-existing pulmonary disease to develop this type of histoplasmosis [1], which is evident in our 39-year-old non-smoker patient. We suggest that a combination of genetic and environmental factors contributed to the development of chronic cavitary histoplasmosis in our patient, possibly owing to cytokine signaling disruption particularly involving interleukin-1, interleukin-2, interferon-gamma, and TNF-a, this could have caused a transient immunocompromised state leading to ineffective granuloma formation and maintenance with consequent dissemination of the organism, dysregulated host inflammatory response, and chronic parenchymal lung disease with cavitary destruction.

**Table 1:** Overview of histoplasmosis syndrome, clinical presentation, and management * Can occur as primary or secondary manifestation.

Type	Involvement	Clinical features	Diagnostics	Prognosis
Primary*	Pulmonary [12,13]	Asymptomatic Fever, Cough Night sweats Weight loss	Solitary calcified lesions or calcified hilar/mediastinal nodes on imaging. Can manifest as solitary endo- bronchial lesions. Definitive diagnosis with bronchoalveolar lavage.	Usually resolve with treatment in immunocompetent patients. Can evolve into disseminated in immunocompromised patients.
	Oral [14]	Palatal, gingival, or tongue ulcerations	Diagnosed with biopsy, suspected in cases of disseminated histoplasmosis but can manifest alone as primary disease.	
	Laryngeal [15]	Progressive hoarseness		
	Cutaneous [16]	Disseminated multiple papulo-nodular lesions.		
	Gastrointestinal [17,18]	Abdominal pain, diarrhea, hepatosplenomegaly, digestive complications.		
Disseminated	Central nervous system [18,19]	Neurological deficits (aphasia, dysarthria, eye motor deficits, motor weakness, and others)	Multiple ring-enhancing lesions on MRI.	Good response to treatment with systemic antifungals
	Bone marrow [20]	Pancytopenia	Bone marrow aspiration reveals maturation arrest, plasmacytosis, and rounded fungal structures inside histiocytes. Definitive diagnosis with a biopsy.	
	Adrenal glands [21]	Adrenal insufficiency (syncope, hypotension, electrolytes imbalance, abnormal pigmentation)	Computed tomography shows bilateral adrenal enlargement with heterogeneous enhancement (mimic metastases). Definitive diagnosis with biopsy.	
Chronic	Chronic cavitary histoplasmosis [22]	Most commonly Cough, Chest pain, Dyspnea, Hemoptysis, Weight loss, Fatigue and Sputum production,	Computed tomography shows dense, sharply demarcated area of consolidation with translucent pockets “moth eaten or Swiss cheese”–like appearance. About 98% of patients manifest with upper lobe cavitation and more often on the right apex.	Complications may occur including mediastinal fibrosis, histoplasmoma and broncholithiasis. Good response to antifungals
	Histoplasma nodules [22]	Usually asymptomatic. The most common feature of chronic pulmonary aspergillosis	Computed tomography shows isolated nodules. Mimic malignancy	The main long-term manifest of histoplasmosis in immunocompetent patients

Cavitary histoplasmosis presents with fatigue, fever, night sweats, anorexia, and weight loss. More specific respiratory symptoms include cough, sputum production, hemoptysis, and shortness of breath, which can mimic a chronic obstructive pulmonary disease (COPD) exacerbation in these patients. The differential diagnosis includes primarily TB, as the presentation is very similar, with chest imaging demonstrating large cavitary lesions with fibrosis. A characteristic feature of chronic cavitary histoplasmosis is the “marching cavity”, where the cavities progress in size, due to continuing necrosis, to involve the entire lobe of the lung [1].

Other differential diagnoses include non-tuberculous mycobacterial infection, particularly *Mycobacterium avium* complex and *Mycobacterial kansasii*; other endemic fungal infections like blastomycosis, coccidioidomycosis, sporotrichosis, and lastly sarcoidosis [1].

Fibrosing mediastinitis is a rare but fatal complication of pulmonary histoplasmosis, usually occurring in young adults aged 20-40 years, with a slight preponderance in women [1]. It has a reported prevalence of three per 100,000 cases [23]. The pathogenesis involves an exaggerated production of fibrous tissue with an encasing of the mediastinal structures, such as the pulmonary vessels, superior vena cava, and airways. It is an idiosyncratic reaction to *Histoplasma* antigens that occurs in a particular group of patients, which insinuates a possibly abnormal immunological host response in these patients to *Histoplasma* infection [1,23].

**Figure 3:**
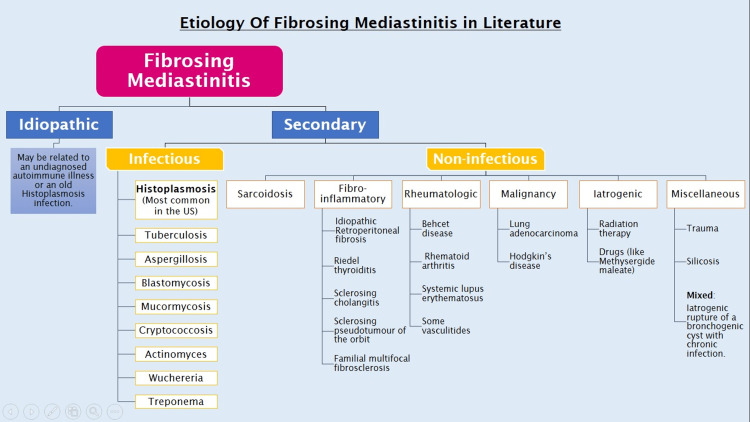
Causes of fibrosing mediastinitis

Reviewing the literature on the etiology of fibrosing mediastinitis, it is evident that wide variations exist throughout different geographical locations. For instance, a recent review showed that Histoplasmosis, as an underlying cause, has incidences of 0-83% in different studies [24]. Figure 3 summarizes the documented causes of fibrosing mediastinitis. 

The symptoms are due to the slowly progressive encroachment of mediastinal structures, and present as increasing dyspnea, cough, hemoptysis, and chest pain, without the systemic signs of infection such as fever, chills, and night sweats [1]. The diagnosis is made clinically and radiographically, and by excluding malignancy, radiation therapy, and thromboembolic disease [23]. F-fluorodeoxyglucose positron emission tomography/computed tomography (FDG-PET/CT) scan commonly shows heightened metabolic activity in foci with fibrosing mediastinitis [25]. It is worthwhile to note that Westerley et al. utilized off-label rituximab therapy, in conjunction with prednisone at the time of the infusion, in three patients with documentation of chronic inflammation and fibrosis on biopsy, and evidence of increased metabolic activity on FDG-PET/CT. All three patients had a favorable therapeutic outcome with clinical improvement and minimization of the fibrous tissue burden, as well as reduction of the metabolic activity on PET/CT scan. Consequently, it was hypothesized that B lymphocytes might play a role in the evolution of fibrosing mediastinitis and are associated with the increased metabolic activity visible on FDG-PET/CT scan. The use of rituximab can attenuate the associated metabolic activity, prevent further disease progression, and ameliorate clinical symptoms [25].

Our patient’s repeat CT revealed signs of vascular involvement suggestive of early fibrosing mediastinitis. The gold standard for diagnosing histoplasmosis is culture on Sabouraud agar demonstrating the organism, with a sensitivity of 67% in chronic pulmonary histoplasmosis. A more rapid diagnosis can be made by histopathology of the biopsy from the affected tissue revealing necrotizing granulomas, with a sensitivity of 75%. In the majority of chronic pulmonary histoplasmosis cases, isolation of *Histoplasma* from bronchoscopy and sputum samples can confirm the diagnosis. Moreover, *Histoplasma* can be detected in bronchial lavage washings. Antigen testing in urine and/or serum is usually negative due to the low burden of the organism, while serologic testing is positive in almost all cases and provides the cornerstone for diagnosis in up to one-quarter of the cases [5]. The most commonly used serologic tests are immunodiffusion (ID) and complement fixation (CF), with a reported sensitivity of 70% in patients with culture-confirmed histoplasmosis [26]. This is in comparison to enzyme immunoassay (EIA), which is of lower sensitivity, cost, and labor intensity compared to CF and ID. Noticeably, both the sensitivity and specificity of CF and ID are influenced by the patient population being tested, the reagents used during testing, and the specific technique used by laboratory personnel [26].

**Figure 4:**
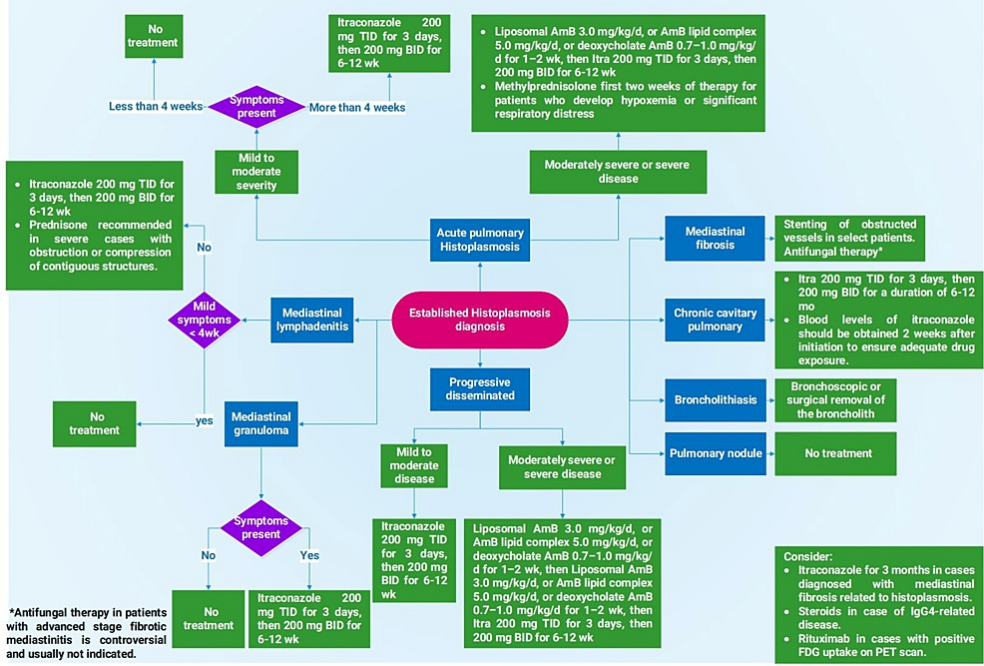
Therapeutic flowchart for histoplasmosis syndrome Adapted From:  Wheat et al., 2007 [27]

Antifungal treatment is mandated for all cases of chronic pulmonary histoplasmosis to decrease the mortality and to halt the progression of the disease, unlike most of the other histoplasmosis syndrome presentations in which observation is the best-recommended approach. Figure 4 illustrates the different treatment regimens for each histoplasmosis syndrome according to the 2007 update of the Infectious Diseases Society of America clinical practice guidelines [27]. In regard to CCPH, the recommended treatment duration is controversial; however, updated guidelines recommend therapy with itraconazole for 12-24 months, and/or until no additional improvement is noticeable on CT. Follow-up is advised for one to two years after treatment is discontinued as 10-20% of the cases relapse off therapy [5]. 

## Conclusions

We described a case of CCPH in a young immunocompetent patient without a smoking history, who showed early signs of fibrosing mediastinitis. Although CCPH generally affects immunocompetent individuals with underlying structural lung disease, it can infrequently present in the absence of an underlying structural lung pathology. The pathogenesis of CCPH involves defects in cellular-mediated immunity and granuloma formation; however, how it affects healthy immunocompetent individuals without underlying lung disease remains an enigma. The treatment duration for CCPH is controversial, nonetheless, a prolonged duration with antifungal treatment is recommended, in addition to strict follow-up to detect disease recurrence upon discontinuation of the medication. It is important to emphasize that untreated or partially treated cases often progress to marching cavitary disease with panlobular involvement and/or fibrosing mediastinitis, a rare but fatal complication. Rituximab has demonstrated promising outcomes in the management of fibrosing mediastinitis, which should be explored further by conducting randomized controlled trials.

## References

[1] Kauffman CA (2007). Histoplasmosis: a clinical and laboratory update. Clin Microbiol Rev.

[2] Kauffman CA (2003). Histoplasmosis. Clinical Mycology.

[3] Kataria YP, Campbell PB, Burlingham BT (1981). Acute pulmonary histoplasmosis presenting as adult respiratory distress syndrome: effect of therapy on clinical and laboratory features. South Med J.

[4] Goodwin RA Jr, Owens FT, Snell JD, Hubbard WW, Buchanan RD, Terry RT, Des Prez RM (1976). Chronic pulmonary histoplasmosis. Medicine (Baltimore).

[5] Hage CA, Azar MM, Bahr N, Loyd J, Wheat LJ (2015). Histoplasmosis: up-to-date evidence-based approach to diagnosis and management. Semin Respir Crit Care Med.

[6] Bahr NC, Antinori S, Wheat LJ, Sarosi GA (2015). Histoplasmosis infections worldwide: thinking outside of the Ohio River valley. Curr Trop Med Rep.

[7] Bauddha NK, Jadon RS, Mondal S, Vikram NK, Sood R (2018). Progressive disseminated histoplasmosis in an immunocompetent adult: A case report. Intractable Rare Dis Res.

[8] Newman SL (2001). Cell-mediated immunity to Histoplasma capsulatum. Semin Respir Infect.

[9] Samaddar A, Sharma A, Kumar Ph A, Srivastava S, Shrimali T, Gopalakrishnan M, Bohra GK (2019). Disseminated histoplasmosis in immunocompetent patients from an arid zone in Western India: a case series. Med Mycol Case Rep.

[10] Armstrong PA, Jackson BR, Haselow D (2018). Multistate epidemiology of histoplasmosis, United States, 2011-2014. Emerg Infect Dis.

[11] Chumpangern W, So-Ngern A, Reechaipichitkul W, Meesing A, Ratanawatkul P, Arunsurat I, Chaisuriya N (2021). Presentations of chronic cavitary pulmonary histoplasmosis mimic infected cystic bronchiectasis in an immunocompetent host: a case report. Respir Med Case Rep.

[12] Tobón AM, Gómez BL (2021). Pulmonary histoplasmosis. Mycopathologia.

[13] Takahashi K, Sasaki T, Nabaa B, van Beek EJ, Stanford W, Aburano T (2012). Pulmonary lymphatic drainage to the mediastinum based on computed tomographic observations of the primary complex of pulmonary histoplasmosis. Acta Radiol.

[14] Kamboj M, Rathee R, Narwal A, Devi A, Gupta A, Sivakumar N (2022). Primary oral histoplasmosis in immunocompetent host: case series with review of literature. Indian J Otolaryngol Head Neck Surg.

[15] Gupta DK, Tanwar D, Patel B, Singh V (2022). Laryngeal histoplasmosis: masquerading malignancy. BMJ Case Rep.

[16] Batista JM, Martins MA, Bertollo CM (2021). Primary cutaneous histoplasmosis difficult to treat in immunocompetent patient: case report and literature review. Einstein (Sao Paulo).

[17] Nacher M, Valdes A, Adenis A (2021). Gastrointestinal disseminated histoplasmosis in HIV-infected patients: a descriptive and comparative study. PLoS Negl Trop Dis.

[18] McGrath M, Nguyen R, Tyrtova E, Ravanpay AC (2022). Reactivation of disseminated histoplasmosis with central nervous system involvement following a primary gastrointestinal histoplasmosis infection: a case report. Cureus.

[19] Hariri OR, Minasian T, Quadri SA, Dyurgerova A, Farr S, Miulli DE, Siddiqi J (2015). Histoplasmosis with deep CNS involvement: case presentation with discussion and literature review. J Neurol Surg Rep.

[20] Lage LA, Lima GG, Groetares de Lima G, Culler HF, Pereira J (2022). Disseminated histoplasmosis and erythrophagocytosis in an immunocompromised host: the role of bone marrow evaluation for prompt diagnosis of invasive fungal infections. Int J Infect Dis.

[21] Robinson LJ, Lu M, Elsayed S, Joy TR (2019). Bilateral adrenal histoplasmosis manifesting as primary adrenal insufficiency. CMAJ.

[22] Baker J, Kosmidis C, Rozaliyani A, Wahyuningsih R, Denning DW (2020). Chronic pulmonary histoplasmosis-a scoping literature review. Open Forum Infect Dis.

[23] Strock SB, Gaudieri S, Mallal S (2015). Fibrosing mediastinitis complicating prior histoplasmosis is associated with human leukocyte antigen DQB1*04:02 - a case control study. BMC Infect Dis.

[24] Kobayashi Y, Ishiguro T, Takaku Y, Kagiyama N, Shimizu Y, Takayanagi N (2021). Clinical features of fibrosing mediastinitis in Japanese patients: two case reports and a literature review. Intern Med.

[25] Westerly BD, Johnson GB, Maldonado F, Utz JP, Specks U, Peikert T (2014). Targeting B lymphocytes in progressive fibrosing mediastinitis. Am J Respir Crit Care Med.

[26] Fida M, Misra A, Harring JA, Kubbara A, Theel ES (2022). Histoplasma capsulatum complement fixation and immunodiffusion assay sensitivity in culture-confirmed cases of histoplasmosis: a 10-year retrospective review (2011 to 2020). J Clin Microbiol.

[27] Wheat LJ, Freifeld AG, Kleiman MB, Baddley JW, McKinsey DS, Loyd JE, Kauffman CA (2007). Clinical practice guidelines for the management of patients with histoplasmosis: 2007 update by the Infectious Diseases Society of America. Clin Infect Dis.

